# Chromosomal Bands Affected by Acute Oil Exposure and DNA Repair Errors

**DOI:** 10.1371/journal.pone.0081276

**Published:** 2013-11-26

**Authors:** Gemma Monyarch, Fernanda de Castro Reis, Jan-Paul Zock, Jesús Giraldo, Francisco Pozo-Rodríguez, Ana Espinosa, Gema Rodríguez-Trigo, Hector Verea, Gemma Castaño-Vinyals, Federico P. Gómez, Josep M. Antó, Maria Dolors Coll, Joan Albert Barberà, Carme Fuster

**Affiliations:** 1 Unitat de Biologia Cel·lular i Genètica Mèdica, Facultat de Medicina, Universitat Autònoma de Barcelona (UAB), Bellaterra, Spain; 2 Centre de Recerca en Epidemiologia Ambiental (CREAL), Barcelona, Spain; 3 Institut de Recerca Hospital del Mar (IMIM), Barcelona, Spain; 4 CIBER Epidemiologia i Salut Pública (CIBERESP), Barcelona, Spain; 5 Unitat de Bioestadística and Institut de Neurociències, Facultat de Medicina, UAB, Bellaterra, Spain; 6 Departamento de Medicina Respiratoria, Unidad Epidemiologia Clínica, Hospital 12 de Octubre, Madrid, Spain; 7 CIBER Enfermedades Respiratorias (CIBERES), Bunyola, Mallorca, Spain; 8 Departamento de Medicina Respiratoria, Hospital Clínico San Carlos, Madrid, Spain; 9 Departamento de Medicina Respiratoria, Complexo Hospitalario Universitario A Coruña, A Coruña, Spain; 10 Departament de Medicina Respiratòria, Hospital Clínic-Institut d’Investigacions Biomèdiques August Pi i Sunyer (IDIBAPS), Barcelona, Spain; 11 Departament de Ciències Experimentals i de la Salut, Universitat Pompeu Fabra (UPF), Barcelona, Spain; 12 Unitat de Biologia Cel·lular, Facultat de Ciències, UAB, Bellaterra, Spain; University of Hawaii Cancer Center, United States of America

## Abstract

**Background:**

In a previous study, we showed that individuals who had participated in oil clean-up tasks after the wreckage of the Prestige presented an increase of structural chromosomal alterations two years after the acute exposure had occurred. Other studies have also reported the presence of DNA damage during acute oil exposure, but little is known about the long term persistence of chromosomal alterations, which can be considered as a marker of cancer risk.

**Objectives:**

We analyzed whether the breakpoints involved in chromosomal damage can help to assess the risk of cancer as well as to investigate their possible association with DNA repair efficiency.

**Methods:**

Cytogenetic analyses were carried out on the same individuals of our previous study and DNA repair errors were assessed in cultures with aphidicolin.

**Results:**

Three chromosomal bands, 2q21, 3q27 and 5q31, were most affected by acute oil exposure. The dysfunction in DNA repair mechanisms, expressed as chromosomal damage, was significantly higher in exposed-oil participants than in those not exposed (p= 0.016).

**Conclusion:**

The present study shows that breaks in 2q21, 3q27 and 5q31 chromosomal bands, which are commonly involved in hematological cancer, could be considered useful genotoxic oil biomarkers. Moreover, breakages in these bands could induce chromosomal instability, which can explain the increased risk of cancer (leukemia and lymphomas) reported in chronically benzene-exposed individuals. In addition, it has been determined that the individuals who participated in clean-up of the oil spill presented an alteration of their DNA repair mechanisms two years after exposure.

## Introduction

In 2002, the oil tanker *Prestige* foundered and spilled more than 67,000 tons of the tanker´s oil, which contaminated more than 1,000 km of the coast of Galicia (North-West Spain). In response more than 300,000 clean-up workers were mobilized. The fact that the oil had a high content of aromatic hydrocarbons (50% by weight), saturated hydrocarbons, heavy metals, resins and asphaltenes, which are classified by the International Agency for Research on Cancer [1] as carcinogens or potential/probable carcinogens, alerted the scientific community to the value of investigating the genotoxic effects on human exposure to the *Prestige* oil. 

Genotoxic studies conducted on populations exposed to the clean-up of oil spills are scarce [2-10]. Two of these studies had been performed before the *Prestige* accident [2,3], and seven more were performed on clean-up workers of the *Prestige* oil spill [4-10]. Different types of biomarkers were analyzed to address the potential genotoxic effects of acute oil-exposure research. DNA adducts were analyzed by Cole et al. [3], while sister chromatid exchanges, micronucleus and comet assay tests were used as biomarkers by others [4-9], while only two groups analyzed chromosomal damage [2,10]. Not all of the biomarkers analyzed showed significant differences between exposed and non-exposed individuals, although in the majority of them, increased genomic damage in exposed individuals has been documented. Moreover, most of these studies were carried out during the oil exposure [2-9]. No information is available regarding the reversibility or persistence of the negative oil effects. So far, only our study, reported by Rodríguez-Trigo et al. [10] has revealed an increase of chromosomal alterations (CAs) in circulating lymphocytes in exposed individuals two years after oil exposure. The findings were unexpected, due to the long time-period that had passed following exposure, and relevant due to the fact that a high number of CAs is associated with a higher risk of developing cancer, as described in the literature [11-15]. These observations led us to make a complete cytogenetic study of the same individuals.

The aim of the present study is to identify if there are specific chromosomal regions especially affected by oil exposure in the same chromosomal preparations of individuals in which an increase of chromosomal damage was found [10]. In addition, we also determined the possible existence of errors in DNA repair mechanisms by analyzing the chromosomal damage in cultures with aphidicolin, an inhibitor of DNA polymerase α and other polymerases.

## Materials and Methods

### Study population

In this study, an accurate selection of individuals highly exposed to the oil was performed [10,16]. Only slightly over 1% (137/10,000) of the individuals, who were non-smokers and had been initially invited, were included in the study. The collection of the samples was performed between 22 to 27 months after the *Prestige* disaster. The project was approved by the Ethics Committee on Clinical Research of Galicia and all participants provided written informed consent. 

### Cytogenetic analysis

Peripheral blood (PB) was cultured in supplemented RPMI-1640 medium (GIBCO Invitrogen Cell Culture, Invitrogen; Carlsbad, California) and then harvested according to standard procedures. For the study of chromosomal bands, PB standard culture at 37°C for 72h was used. The cytogenetic banded preparations previously studied in 91 exposed and 46 non-exposed individuals [10] were re-examined for an accurate identification of breakpoints involved in chromosomal damage. 

For the study of DNA repair efficiency, PB obtained in the same extraction was cultured at 37°C for 96h, and aphidicolin (Sigma Aldrich), an inhibitor of DNA polymerase α and other polymerases, was added to the cultures 24h before harvesting at a final concentration of 0.2µM. The cellular suspension was keep frozen until cytogenetic results without aphidicolin were obtained. Dysfunction in DNA repair mechanisms was studied only in randomly selected female subsamples because women were more prevalent than man is both the samples of exposed and non-exposed individuals [10]. A total of 14 exposed and 14 non-exposed individuals were studied and compared with standard culture without aphidicolin. Chromosomal preparations were uniformly stained with Leishman (1:4 in Leishman buffer) to detect chromosomal damage, expressed mainly as chromosomal lesions (gaps and breaks). Moreover, apparent or large structural CAs (rings, marker chromosomes, dicentric translocations, etc.) were also detected. In these cases, a posterior G banding technique was applied in order to clarify if the apparent CAs were a marker chromosome, a reciprocal translocation, a duplication, etc. A minimum of 100 metaphases were analyzed in each participant according to conventional criteria. 

Criteria for cytogenetic evaluations were determined according to the International System for Human Cytogenetic Nomenclature [17]. 

### Statistical analysis

To identify which chromosomal bands were involved in chromosomal damage using standard culture, two statistical methods were used. First, the Fragile Site Multinomial method, FSM version 995, [18-20], specifically used to determine chromosomal regions with a greater propensity to break. Second, a chi-square test was performed to test the null hypothesis of a uniform distribution among the chromosomal bands, where the bands were corrected by their length. The relative length of the affected bands in relation to total genome was estimated using the diagram of the standardized human karyotype [17]. *A generalized* estimating equation, GEE [20,21], was used for assessing the differences between the exposed and non-exposed groups for the different types of chromosomal damage induced by aphidicolin. The GEE approach is an extension of generalized linear models designed to account for repeated, within-individual measurements. This technique is particularly indicated when the normality assumption is not reasonable, as happens, for instance, with discrete data. The GEE model was used instead of the classic Fisher exact test because the former takes into account the possible within-individual correlation, whereas the latter assumes that all observations are independent. Since several metaphases were analyzed per individual, the GEE model is more appropriate. Statistical significance was set at p< 0.05. Statistical analyses were carried out with SAS/STAT release 9.02 (SAS Institute Inc; Cary, NC). The GEE model was fitted using the REPEATED statement in the GENMOD procedure. The conservative Type 3 statistics score was used for the analysis of the effects in the model.

## Results

### Chromosomal bands most affected by oil exposure

**Figure 1:**
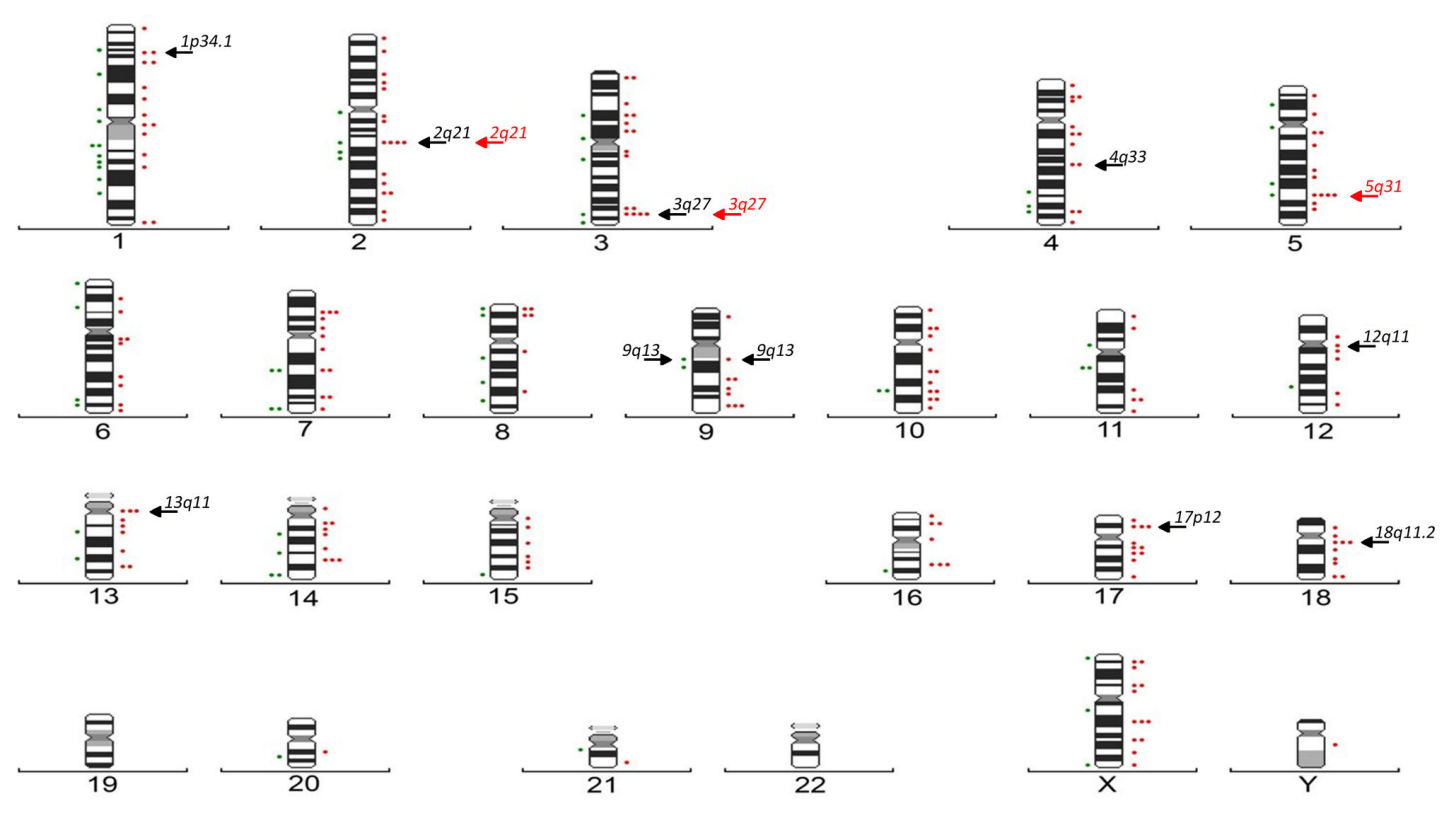
Distribution of chromosome breakpoints observed in exposed (right) and non-exposed individuals (left) in the human ideogram (400-band resolution). The most affected bands using the FSM statistical method are indicated by red arrows, and when using another statistical method that takes into account the relative length of chromosomal bands, by black arrows.

A total of 9,520 and 4,859 metaphases from standard culture were analyzed in 91 exposed and 46 non-exposed individuals, respectively. Table 1 shows the same results described in our previous report [10] using the conventional cytogenetic frequencies in order to compare them with other genotoxic studies. A total of 203 breakpoints in exposed and 61 in non-exposed individuals involved in chromosomal damage (lesions and structural CAs) were detected. The breakpoints distribution in the human ideogram (at the 400-band resolution level) is shown in Figure 1. The breakpoints involved in the chromosomal damage were mainly located on chromosomes 3, 10, 17 and 18 in exposed individuals (*vs.* 1, 8 and 14 in non-exposed). To identify those chromosomal bands that significantly expressed breakpoints in exposed and non-exposed participants, two statistical methods were used. In the first one, the FSM method, the number of breaks required to consider a band as being non-randomly affected was four or more. The most affected bands in the exposed group were 2q21, 3q27 and 5q31 (*vs.* none in non-exposed). On the other hand, the second statistical method, using the chi-square test, was applied considering the relative length of chromosomal bands identified as 1p34.1, 2q21, 3q27, 4q33, 9q13, 12q11, 13q11, 17p12 and 18q11.2 bands in exposed *vs* the 9q13 band in non-exposed individuals. It is interesting to note that only 2q21 and 3q27 were considered to be bands affected by both methods.

**Table 1:** Frequency and types of chromosomal damage observed in standard culture.

	**Exposed**	**Non-Exposed**
Total individuals, No.	91	46
Total metaphases analyzed (uniform stain), No.	9520	4859
Total metaphases karyotyped (G-banded), No.	2448	1285
***Chromosomallesion*** (uniform stain), No. (%)	100/9520 (1.05)	35/4859 (0.72)
Gaps	48 (0.5)	19 (0.39)
Breaks	52 (0.55)	16 (0.33)
***Structuralchromosomalalterations*** (G-banded), No. (%)	196/2448 (8)	33/1285 (2.56)
Balanced	12/196 (6.1)	7/33 (21.2)
Reciprocal translocations	10	7
Robertsonian translocations	2	0
Imbalanced	184/196 (93.9)	26/33(78.8)
Deletions	23	6
Deletions + acentric fragments	9	3
Acentric fragments	42	0
Imbalanced translocations	23	3
Dicentric translocations	3	0
Dicentric translocations+acentric fragment	4	1
Rings	9	0
Markers	68	13
Additional material of unknown origin	2	0
Isochromosomes	1	0
***Totalbreakpointsidentified***	203	61
Chromosomal lesion (after G-banded)	98	34
Structural chromosomal alterations (G-banded)	109	27

### DNA repair efficiency

Table 2 shows the chromosomal damage detected using uniform staining in cultures with aphidicolin and standard culture from the same exposed and non-exposed individuals. A total of 1,441 and 1,410 metaphases from cultures with aphidicolin were analyzed in 14 exposed and 14 non-exposed participants, respectively. The chromosomal damage induced by aphidicolin is usually expressed by chromosomal lesions. The number of chromosomal lesions was higher and statistically significant in exposed *vs.* non-exposed individuals (p= 0.023), and almost the same findings were observed in relation to apparently structural CAs (p= 0.024). Chromosomal damage (lesions and structural CAs) was also statistically higher in exposed than in non-exposed individuals (p= 0.016). Due to the large number of chromosomal lesions induced by aphidicolin, their average number is shown per 100 metaphases in Table 2.

**Table 2:** Chromosomal damage observed in E and NE individuals using uniform staining in cultures with aphidicolin *vs* standard culture.

	Culture with aphidicolin								Standard culture				
	Exposed		Non-Exposed			*p*-value			Exposed	Non-Exposed			*p*-value
*Total individuals*	14		14						14	14			
Total metaphases analyzed	1441		1410						1500	1463			
Total metaphases with lesions (%)	947/1441 (65.7)		699/1410 (49.6)			0.0141			12/1500 (0.8)	8/1463 (0.5)			0.4775
Total metaphase with structural alterations No./total (%)	46/1441 (3.2)		16/1410 (1.1)			0.0376			15/1500 (0.01)	1/1463 (0.07)			0.0594
***Chromosomallesions*** (% in 100 cells)	1864/1441 (129.3)		1216/1410 (86.2)			0.0231			13/1500 (0.9)	10/1463 (0.7)			0.6455
Gaps	1007		623						6	6			
Chromatid gap	496		346						5	6			
Chromosome gap	511		277						1	0			
Breaks	857		593						7	4			
Chromatid break	239		107						5	2			
Chromosome break	618		487						2	2			
***Apparent structural chromosomal alterations* (%)**	63/1441 (4.4)		19/1410 (1.4)			0.0239			24/1500 (1.6)	1/1463 (0.07)			0.0791
Balanced													
Translocations	7		2						0	0			
Imbalanced													
Deletions	12		0						0	0			
Acentric fragments	11		3						16	1			
Imbalanced translocations	9		0						0	0			
Dicentric translocations	15		12						0	0			
Rings	7		1						2	0			
Markers	1		1						6	0			
Duplications	1		0						0	0			
**Total chromosomal damage (%)**	1927/1441 (1.33)		1235/1410 (0.87)			0.0161			37/1500 (0.025)	11/1500 (0.07)			0.1080

## Discussion

A previous study reported by our group [10] has revealed an increase of structural CAs in circulating lymphocytes in exposed individuals two years after the *Prestige* oil exposure. The present study, carried out on the same individuals, shows that a few chromosomal bands exist in the human genome which are particularly sensitive to breakage in acute oil exposure. Furthermore, we also found that the increase in chromosomal damage is due to the existence of a statistically significant reduction of DNA repair efficiency in exposed as compared to non-exposed individuals. 

Structural CAs are considered to be a highly informative biomarker for detecting adverse health effects, such as the risk of developing cancer [11,13,14]. Individuals chronically exposed to benzene have a 20-fold increased risk of developing cancer compared with the general population, particularly hematologic cancer [22-24]. Additionally, an increase in chromosomal damage, especially imbalanced structural CAs, is crucial in the development of cancer [23]. Several authors have suggested that CAs could be used as a biomarker in cancer initiating events [11,12,14]. For this reason, we proposed to determine if some chromosome bands were especially affected by oil exposure and if the effects upon the genes located in these genomic regions, could explain the cellular disorders involved in cancer. To our knowledge, this is the first study concerning chromosomal breakpoints distribution on the human ideogram in relation to acute oil exposure. This distribution was not uniform, with the 2q, 3p, 5q, 10q, 16p, 17p, 18q chromosomal arms being the most affected in exposed individuals. In two previous works only chromosomes involved in breakages were studied revealing that chromosomes 2, 4 and 7 [25] and chromosomes 5 and 7 [26] were the most frequently affected in chronically benzene-exposed individuals. These findings support the hypothesis that the 2q and 5q regions are targets in both acute and chronic exposure.

Our results show that the 2q21 and 3q27 bands are especially affected in exposed individuals when both the FSM method and an additional method that takes into account the relative bands’ lengths were used, while 5q31 was considered affected only when the FSM method was employed. These bands correspond to regions where fragile sites (FRA2F, FRA3C and FRA5C) are located according to the human genome browsers like NCBI (http://www.ncbi.nlm.nih.gov/). Although not all fragile sites may be equally involved in cancer development, it is known that they are vulnerable targets for various oncogenic agents, and their damage may potentially produce consequences for genomic integrity [27]. There is evidence that these fragile sites are involved *in vivo* in CAs related to tumor development [28]. According to information available from human genome browsers like NCBI (http://www.ncbi.nlm.nih.gov/), in these bands we can find genes involved in different cellular processes (Table 3) such as cellular cycle control (*CCNT2* at 2q21; *CDC25C*, *CDC23* and *CDKL3* at 5q31), DNA repair (*ERCC3* at 2q21), proto-oncogenes (*CH-Ras* at 2q21; *TGFBI* at 5q31), tumor suppressor genes (*CXCR4, NMTC1* and *TP21* at 2q21; *BCL6* at 3q27; *IRF1*and *EGR1* at 5q31), and one gene is involved in the apoptotic process (*DND1* at 5q31). Results of the present study support the hypothesis that the oil components, some of them considered to be carcinogenic, may induce mutations, mainly due to breakages in specific chromosome regions (2q21, 3q27, 5q31). The 11q23 band, while especially sensitive to benzene and commonly involved in hematopoietic malignance [22,29,30], was not considered statistically significant in our study, as it was observed only in two CAs in exposed individuals. To some extent such changes, when accumulated, may cause deletions or disruptions of functional genes, thus increasing the risk of cancer, cannot be deduced from the present study. It is of interest to note that much of chromosomal reorganization in hematopoietic pathologies is associated with the same chromosome bands most affected by exposure to oil (Table 3). For example, patients with T-Cell lymphoma present CAs involving 2q21 and 3q27, acute lymphoblastic leukemia presents 5q31 as a specific chromosome region and acute myeloid leukemia is characterized by chromosomal reorganization at 5q31 and 11q23 bands [31-33]. Our findings show that there are a few chromosomal bands especially prone to breakage in oil exposure that could induce chromosomal instability, which could explain the increased risk of cancer (leukemia and lymphomas) reported in chronically benzene-exposed individuals [22-24]. Nevertheless, future genotoxic studies using the new microarray technologies applied to the genome itself (duplications, deletions, epigenetic changes) and to mRNA translation and its control mechanisms through miRNA are necessary to elucidate the role of genomic instability in the formation of cancer-specific CAs, and impact of environmental factors like oil exposure on this instability. 

**Table 3:** Chromosomal bands most affected in present study, genes and recurrent chromosomal reorganization present in hematopoietic malignances, and comparising with chronic benzene exposure.

**Chromosomal Bands**	**Cellular cycle control**	**DNA repair**	**Oncogenes**	**Tumor suppressors**	**Apoptosis**	**Type of Cancer**	**Individuals exposed chronically to benzene**	**References**
**2q21**	*CCNT2*	*ERCC3*	*CH-Ras*	*CXCR4*	*-*	T-Cell lymphoma	No	[31]
				*NMTC1, TP21*		Chronic lymphocytic leukemia (CLL)	No	[40]
**3q27**	*-*	*-*	*-*	*BCL6*	*-*	T-Cell lymphoma	No	[31]
						Non-Hodgkin lymphoma (NHL)	No	[41]
**5q31**	*CDC23*	*-*	*TGFBI*	*IRF1*	*DND1*	Acute lymphoblastic leukemia (ALL)	No	[32]
	*CDC25*			*EGR1*		Myelodysplastic Syndrome (MDS)	Yes	[42]
	*CDKL3*					Chronic myelomonocytic leukemia (CMML)	No	[43]
						Myelodysplastic Syndrome	No	[44]
						Acute myeloid leukemia (AML) and myelodysplastic Syndrome (MDS)	No	[45]
						Acute lymphoblastic leukemia (ALL)	No	[46]
**11q23**	-	-	-	-	-	Leukemogenesis (ALL and AML)	No	[47]
						Acute lymphoblastic leukemia (ALL)	No	[32]
						Acute lymphoblastic leukemia (ALL)	Yes	[23]
						Acute lymphoblastic leukemia (ALL)	No	[30]
**5q31/11q23**	-	-	-	-	-	Acute myeloid leukemia (AML)	Yes	[33]

The toxic and carcinogenic compounds of the oil, such as aromatic hydrocarbons, may induce CAs directly or indirectly, by affecting the DNA repair mechanisms, in a process that, if persistent, might predispose cells to the development of cancer. To determine whether the increase in structural CAs previously detected [10] in exposed participants could be a consequence of dysfunctions in the DNA repair mechanisms, aphidicolin was added to the culture media. Aphidicolin is an inhibitor of the DNA polymerases α, δ and ε. These polymerases are involved in DNA replication and repair. The presence of aphidicolin produces breaks in DNA by stopping replication or by causing dysfunctions in DNA repair (nucleotide excision repair and base excision repair) [34]. The breakages induced by aphidicolin could be analysed using several biomarkers such as comet assay, micronucleus testing or chromosomal lesions/structural alterations analysis. The most useful biomarker is chromosomal lesions because the increase of lesions is associated with a mutagenic agent effect [35]. Our results, in 14 exposed and 14 non-exposed individuals, show that the chromosomal damage, expressed mainly as chromosomal lesions, is statistically significant and higher in exposed than in non-exposed participants, confirming the existence of dysfunction in DNA repair mechanisms due to oil exposure. Previous studies using ionizing radiation instead of aphidicolin [36-38] reported that chronically benzene-exposed individuals show a lower DNA repair efficiency. These findings along with the present study suggest that chronic and acute exposure to benzene/oil could affect DNA repair. In this regard, it has recently been published [39] that individuals who have chronic exposure to toxic substances will develop DNA repair deficiency, suggesting that this functional biomarker can be used to predict genetic risk of cancer.

These findings suggest that, in the same way that benzene may induce hematopoietic malignancies, acute oil exposure may be involved in the origin of cancer caused by chromosomal damage. Nonetheless, taking into account the wide inter-individual genetic susceptibility to carcinogens in the general population, more genetic research is necessary to clarify the existence of a relationship between acute oil exposure and subsequent cancer development. Finally, if this relationship is confirmed, this cancer risk cannot be extrapolated to the approximately 300,000 individuals that participated occasionally in oil clean-up tasks because, in our study, exposed individuals were strictly selected on the basis of intense exposure. Additionally, limitations of the present study include the small sample size, and the possibility of some kind of selection bias should be considered.

## Conclusion

Our findings show an increase of chromosomal breakage at 2q21, 3q27 and 5q31 bands in PB lymphocytes two years after exposure. These chromosomal bands, which are commonly involved in hematological cancer, could be considered as useful genotoxic oil biomarkers. Moreover, breakages in these bands could induce chromosomal instability, which can explain the increased risk of cancer (leukemia and lymphomas) reported in chronically benzene-exposed individuals. In addition, our results suggest that the increase in chromosomal damage in these individuals could be a consequence of the reduction of DNA repair efficiency**.**
